# Thromboprophylaxis for Hospitalized Patients with Inflammatory Bowel Disease—Are We There Yet?

**DOI:** 10.3390/jcm9092753

**Published:** 2020-08-26

**Authors:** Asaf Levartovsky, Yiftach Barash, Shomron Ben-Horin, Bella Ungar, Eyal Klang, Shelly Soffer, Uri Kopylov

**Affiliations:** 1 Department of Gastroenterology, Sheba Medical Center, Tel Hashomer, Ramat Gan 5262100, Israel; shomron.benhorin@gmail.com (S.B.-H.); bella.geyshis.ungar@gmail.com (B.U.); ukopylov@gmail.com (U.K.); 2 Sackler Faculty of Medicine, Tel Aviv University, Ramat Aviv 69978, Tel Aviv, Israel; 3 Department of Diagnostic Imaging, Sheba Medical Center, Tel Hashomer, Ramat Gan 5262100, Israel; yibarash@gmail.com (Y.B.); eyalkla@hotmail.com (E.K.); soffer.shelly@gmail.com (S.S.); 4 DeepVision Lab, Sheba Medical Center, Tel Hashomer, Ramat Gan 5262100, Israel

**Keywords:** venous thromboembolism, prophylaxis, inflammatory bowel diseases, extra-intestinal manifestations, hospitalization

## Abstract

Patients with inflammatory bowel disease (IBD) have a high risk of venous thromboembolism (VTE) events in both hospitalized patients and outpatients. Although thromboprophylaxis is recommended for hospitalized patients with IBD, implementation is not universal, especially for non IBD-related hospitalizations. Our objective was to present VTE and thromboprophylaxis adherence rates among hospitalized patients with IBD. An electronic data repository was created of all patients with IBD who visited the emergency department (ED) of our tertiary medical center between 2012 and 2018. The data included tabular variables and free-text physician records. We searched the data for VTE events, using ICD10 coding. Overall, there were 7009 ED visits of 2405 patients with IBD, 1556 (64.7%) with Crohn’s disease (CD) and 849 (35.3%) with ulcerative colitis (UC). Thromboprophylaxis was administered in 463 hospitalizations (12.4% of IBD-related and 10.9% of non IBD-related hospitalizations, *p* = 0.13). Nineteen VTEs were diagnosed in the ED and seventeen were diagnosed during hospitalization (11 non IBD-related and 6 IBD-related hospitalizations, 0.6% and 0.28% respectively, *p* = 0.12). One patient died during hospitalization and an additional two in the 90 days post-discharge from hospitalization (unrelated to VTEs). In conclusion, thromboprophylaxis rates in hospitalized patients with IBD are low, despite possible implications and established guidelines. Thromboprophylaxis should be implemented in patients with IBD hospitalized for all indications.

## 1. Introduction

Inflammatory bowel diseases (IBD) comprise a variety of intestinal and extra-intestinal manifestations. One of the more common extra-intestinal manifestations are thromboembolic complications such as venous thromboembolism (VTE) events [1]. The most common forms of VTEs are deep venous thrombosis (DVT), usually located in the lower extremities, and pulmonary embolism (PE). Less common sites such as cerebrovascular, portal, and mesenteric veins have also been reported [2,3].

Patients with IBD have a greater risk of VTEs compared to the general population [4,5,6,7]. Studies throughout the years, including large meta-analyses, demonstrated a 2-3times higher probability of a VTE event occurring among patients with IBD [8,9]. In IBD, VTE events are often associated with intestinal disease activity, hence their incidence increases during flares [5,10]. Hospitalization is a strong risk factor for the development of a VTE, probably due to the combination of immobility and an acute disease [5,6]. Inpatients who develop VTEs tend to have longer hospitalizations and increased mortality associated with IBD [6]. However, VTE events also tend to occur in the outpatient setting as well, specifically with concurrent disease-related risk factors such as central-venous catheters, immobilization, and recent hospitalization [11,12,13]. Patients with IBD have higher rates of recurrent VTE, especially after the discontinuation of VTE prophylaxis (thromboprophylaxis) [14]. Additional significant risk factors for the development of VTE in patients with IBD include colonic disease in Crohn’s disease (CD) and recent surgery, especially in the prior 14 days [15,16,17]. Complicated disease, characterized by strictures or fistulas, are independently associated with an increased risk of VTE [6].

As the risk for VTE in patients with IBD increases during hospitalization, and pharmacological thromboprophylaxis therapy can lower the risk of VTE post-discharge, there is a consensus to treat all IBD inpatients with thromboprophylaxis [18,19]. However, there is significant variation in the reported rates of thromboprophylaxis in hospitalized patients with IBD among gastroenterologists [20,21,22]. There are still debatable issues regarding prophylactic treatment. There is no consensus for prophylactic treatment of hospitalized patients with non IBD conditions or for outpatients during acute flares of IBD without a history of prior VTE. In addition, the duration of prophylaxis during hospitalization and the impact on subsequent risk of VTE post-discharge remains uncertain.

Data regarding IBD hospitalizations and the risk for VTE events are essential for understanding the overall burden of the disease and the utilization of preventive measures. In this study, we reviewed a large cohort of patients with IBD referred to a large tertiary hospital, and determined the rate and incidence of VTE events. In addition, we examined the adherence to thromboprophylaxis according to established guidelines.

## 2. Materials and Methods

### 2.1. Data Collection of Patients with IBD

We created an electronic data repository of all patients with IBD who visited the emergency department (ED) of the Sheba medical center, a tertiary medical center, between January 2012 to December 2018. Data included tabular demographic and clinical variables, as well as free-text physician records. For this study, we searched the data repository for VTE cases, using ICD10 coding (DVT-I82.9, PE-I26.99).

### 2.2. Cohort IBD Analysis

The cohort was initially analyzed to identify IBD classification, age and gender of patients. For each ED visit, the reason for referral was extracted and classified as IBD-related (abdominal pain, diarrhea, bloody stools, intestinal obstruction) or non IBD-related. A non IBD-related visit was defined as an ED visit due to symptoms different from those stated above (trauma, dyspnea, chest pain, weakness, back pain, limb problems, etc.). This was essential in order to differentiate patients with significant non IBD-related risk factors, such as malignancy and immobility. In addition, it was specified for every patient with IBD who visited the ED whether the patient was hospitalized, in what department and if thromboprophylaxis was administered during hospitalization.

A second analysis was done of patients with IBD who were diagnosed with a DVT and/or PE in the ED or during the current hospitalization. The electronic medical records of these patients were manually reviewed for clinical data that were not included in the original file: IBD details (disease extent, behavior, extraintestinal manifestations, therapy), clinical characteristics, VTE type and location, additional risk factors for VTE, treatment details and hospitalization outcomes. Patients with a diagnosis of superficial thrombophlebitis, superficial thromboembolism or chronic VTE were excluded.

### 2.3. VTE Diagnosis and Treatment

All VTEs were diagnosed and confirmed by standard protocols—ultrasound doppler for DVT of lower and upper extremities, computed tomography (CT) pulmonary angiography for PE and CT for VTE of other locations. Lower extremity DVT was defined as proximal if located in the popliteal, femoral, or iliac veins.

The standard pharmacological prophylactic treatment for VTE in our medical center is enoxaparin, a low-molecular-weight heparin, at a dose of 40 mg subcutaneously once daily [23]. The dosage was modified depending on renal function. Thromboprophylaxis was considered administered during hospitalization if it was given at least once in the absence of a VTE diagnosis. Other agents for the prevention of thromboembolic events, such as warfarin or direct oral anticoagulants, were not considered in this study. In addition, compression stockings are generally not considered for routine thromboprophylaxis in our medical center.

### 2.4. Statistical Analysis

Continuous variables were presented as medians and intra-quartile ranges (IQR) and categorical variables as percentages. VTE and thromboprophylaxis administration rates were correlated with the reason for the ED admission (IBD-related vs. non IBD-related) using the chi-square test and Fisher’s exact test. All statistical tests were two-sided, and a *p* value < 0.05 was considered statistically significant. Statistical analysis was performed using the SPSS v23 statistical software (Armonk, NY, USA).

## 3. Results

### 3.1. Emergency Department Visits of Patients with IBD

Overall, there were 7009 ED visits of 2405 patients with IBD, 1556 (64.7%) with CD and 849 (35.3%) with ulcerative colitis (UC). There were 1216 (50.5%) male patients and 1189 (49.5%) female patients, with an average of 2.91 ED visits per patient during the study period (Table 1).

**Figure 1:**
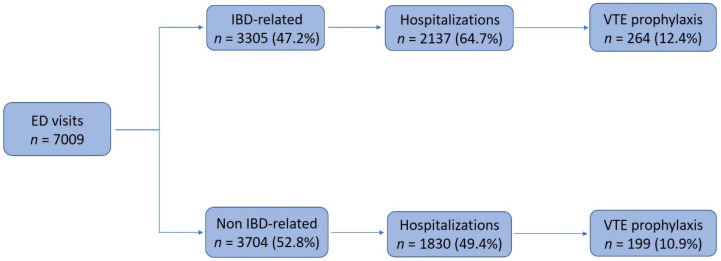
Distribution of all emergency department visits to IBD-related vs. non IBD-related admissions as well as hospitalizations and thromboprophylaxis administration rates.

The median age of the patients was 42 years (IQR 28–65). A total of 3305 (47.2%) ED visits were due to IBD-related complaints. More than half of the ED visits (3967, 56.6%) resulted in a hospitalization, for a median length of 4 days (IQR 2–7). Of those, 1505 (37.9%) and 1510 (38.1%) hospitalizations were in the internal medicine and general surgery departments, respectively (Figure 1).

### 3.2. Prophylactic Anticoagulation during All Hospitalizations

**Figure 2:**
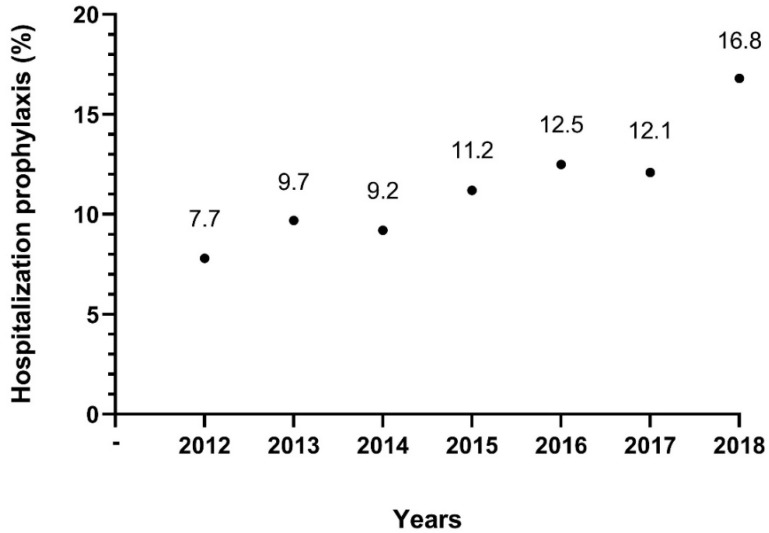
VTE prophylaxis rates of hospitalized patients with IBD between 2012 and 2018.

Thromboprophylaxis was administered in 463 hospitalizations (11.7% of all hospitalizations). The prophylaxis rate was 7.7% (39/505 hospitalizations) in 2012 and 16.8% (118/703 hospitalizations) in 2018 (Figure 2). The prophylactic treatment rate in IBD-related and non IBD-related hospitalizations was 12.4% (264/2137) and 10.9% (199/1830), respectively. There was no significant difference in thromboprophylaxis administration rates between the groups (*p* = 0.13).

### 3.3. Clinical Characteristics of All New VTE Patients

Of the 2405 patients with IBD who visited the ED, 36 (1.5%) were diagnosed with a new VTE (19 in the ED, 17 during hospitalization). There was an equal distribution of gender and IBD type (18 males vs. 18 females; 18 CD vs. 18 UC) and the median age at VTE diagnosis was 64.5 years (IQR 48.25–79.25). Thirty-four cases of deep vein thrombosis and 8 pulmonary embolisms were diagnosed (Table 2). Thirty patients had an isolated VTE event (28 DVT, 2 PE) and six patients were diagnosed with both a DVT and PE. Most cases were defined as proximal DVTs (82.4%), and three patients had a proximal and distal DVT. All patients who were diagnosed with a DVT had initial symptoms of pain or swelling in a unilateral lower limb. A total of eight patients were diagnosed with a PE. The main symptoms on presentation were dyspnea and chest pain (four and two patients, respectively). Two patients had no PE-related symptoms, yet were diagnosed due to an initial diagnosis of a lower limb DVT.

In terms of risk factors, six patients (16.7%) had a medical background of malignancy, four patients (12.5%) were considered bedridden and two (5.55%) had a central venous catheter for total parenteral nutrition (TPN). Six patients (16.7%) were in a post-operative period and VTEs were diagnosed after a median of 12 days post-surgery (IQR 9.25–18). Twenty-one patients (58.3%) did not have thrombosis-related risk factors.

The majority of patients with CD (38.9%) had a non-penetrating and non-stricturing disease, with ileo-colonic involvement. Eight patients (22.2%) had additional extra-intestinal manifestations, besides VTE (Table 2). On admission, 12.1% had fever (4/33), 67.6% were anemic (23/34) and 61.8% had leukocytosis (21/34). The median CRP in the ED was 74.9 mg/dL (IQR 14.075–111.5, normal range 0–5). We compared laboratory markers of patients’ first admission (*n* = 2388) to newly diagnosed VTE events during hospitalization. VTE rates were associated with significantly higher white blood cells (12.2 (IQR 10.2–14.2) vs. 9.5 (IQR 7.3–12.4) median, *p* = 0.007) and higher CRP levels (83.4 (32.6–106.7) vs. 24.7 (5.4–72.8) median, *p* = 0.005) compared to patients who were not diagnosed with a VTE during hospitalization (*n* = 2388). There was no significant association between VTE rates and hemoglobin levels on admission.

In terms of medication administered in the hospital, we compared treatment rates of systemic corticosteroids to newly diagnosed VTEs during patients’ first hospitalization. There was a significantly higher corticosteroid administration rate among patients with IBD who were diagnosed with a VTE during hospitalization (8/17 (42%) vs. 529/2388 (22%), *p* = 0.03) compared to patients with IBD who were not diagnosed with a VTE.

### 3.4. Newly Diagnosed VTE during Hospitalization

**Figure 3:**
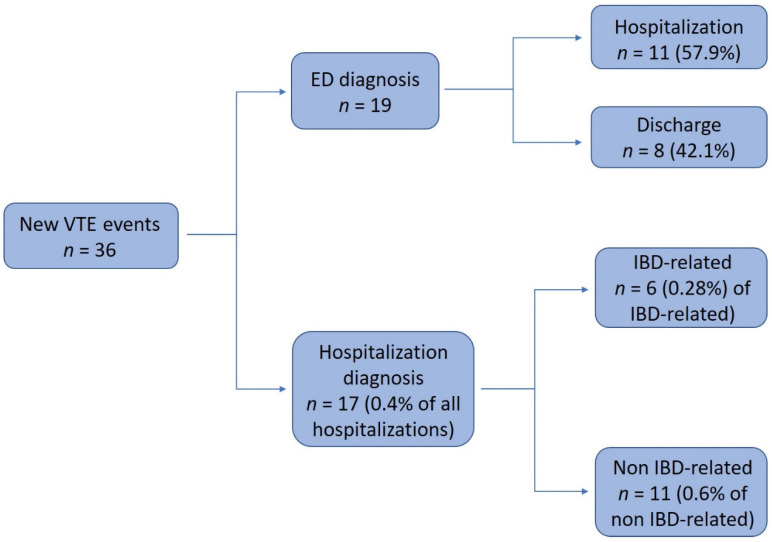
Newly diagnosed VTEs and their distribution between an ED vs. hospitalization diagnosis.

Seventeen patients with IBD (0.4% of total hospitalizations) were diagnosed with a new VTE during hospitalization (Figure 3). The length of hospitalization was a median of 21 days (IQR 7–42.5). Six of the seventeen VTE events were diagnosed during an IBD-related hospitalization (0.28% of all IBD-related hospitalizations) compared to eleven non IBD-related hospitalizations (0.6% of all non IBD-related hospitalizations). There was no significant difference in VTE rates between the groups (*p* = 0.12).

The majority of hospitalizations were in internal medicine departments (64.7%) and a few cases included multiple-department hospitalization (Table 3). In terms of mortality outcome, one patient died during hospitalization with a new diagnosed VTE, another patient died in the next thirty days post-discharge, and an additional patient died in a 90-day period post-discharge from hospitalization. All deaths were of patients who were hospitalized for non IBD-related reasons and were all due to infections (pneumonia (1), septic shock (2)). The median age of death was 82 years.

### 3.5. Prophylactic Anticoagulation during Hospitalizations of VTE Events

Of the six VTE cases diagnosed during an IBD-related hospitalization, thromboprophylaxis was administered to one patient (1/6, 16.6%) following a post-operative period. Four patients (66.6%) were not given anticoagulation at all and one patient was already treated with therapeutic anticoagulation due to a new atrial fibrillation.

In VTE cases diagnosed during a non IBD-related hospitalization, eight patients (8/11, 72.7%) did not have additional thrombosis-related risk factors. Four patients (4/11, 36.4%) were treated with thromboprophylaxis, all due to immobilization. Two patients were administered therapeutic anticoagulation within 24 hours of admission due to clinical suspicion of a DVT. An additional patient was already treated with anticoagulation due to atrial fibrillation and four patients were not administered anticoagulation at all.

Of all the five patients who received thromboprophylaxis during hospitalization (one IBD-related and 4 non IBD-related), three cases were initiated from the first hospitalization day. The median of patient days on thromboprophylaxis prior to the VTE event was 16 (IQR 3–21.5).

### 3.6. Newly Diagnosed VTE in the ED

Nineteen patients with IBD were diagnosed with a new VTE upon arrival to the ED. Of those, eleven patients (11/19 57.9%) were hospitalized for further evaluation and treatment in one of the hospital departments and the others (all with a single DVT) were discharged from the ED with anticoagulation treatment (Figure 3). Almost all of the ED visits (18/19) were due to non IBD-related reasons (the majority–unilateral limb pain). One IBD-related admission (abdominal pain) was diagnosed with an IVC thrombus during a contrast-enhanced CT of the abdomen in the ED.

One patient died within 30 days post-discharge from the ED and another patient in the 90-day post-discharge period. These two cases were of patients who were admitted to the ED for non IBD-related reasons and died from a metastatic malignancy.

### 3.7. Hospitalizations in the Previous 14 Days before VTE Diagnosis

Nine patients (25% of all VTE cases) were hospitalized in the 14 days prior to the diagnosis of VTE (Table S1). The interval between hospitalizations was a median of 8 days (IQR 5–13.5). Of those, three cases were considered IBD exacerbations and two cases were urgent colectomies due to large bowel obstructions secondary to colon carcinomas. These five cases were hospitalized in general surgery departments and the others, all non IBD-related, were hospitalized in internal medicine departments. Two of the nine patients (22.2%) received thromboprophylaxis during their recent hospitalization, and only one of these occurred in an IBD-related admission.

## 4. Discussion

This study described the characteristics of patients with IBD who arrived at the emergency department of a large tertiary hospital. We identified 36 patients who developed a VTE event during a 7-year study period. There was no difference between VTE rates diagnosed during an IBD-related hospitalization and a non IBD-related hospitalization. We showed that thromboprophylaxis administration rates in hospitalized patients with IBD are low, unrelated to their IBD status. It is essential to emphasize that the main purpose of this study was not to establish the efficacy of thromboprophylaxis but rather to evaluate adherence to the established guidelines and administration of thromboprophylaxis adequately.

The increased risk of VTEs in hospitalized patients with IBD is well established in the literature, yet most VTE cases occur in outpatients with risk factors. In this study, 52% of the patients with a new VTE were diagnosed upon arrival to the ED, meaning that they developed VTEs as outpatients. This group of patients had additional risk factors including malignancy (21%), prolonged immobilization (15.8%), and steroid treatment (21%) [24]. Patients with IBD in the post-operative period are at increased risk for post-operative VTE and these tend to form within two weeks, at an average of 10.8 days from surgery [15]. The data in this study are consistent with the literature, as six patients developed VTEs after a median of 12 days post-surgery. These findings only emphasize the need for preventive measures in this at-risk population. Moreover, the VTE cohort was much older (median age of 64.5) compared to non-VTE patients (42), certainly adding age-related risk factors and supporting the literature [25]. There is a tendency for longer hospitalizations when VTE occurs, mainly due to additional risk factors with a high burden of disease. Indeed, in our study, the hospitalization length was longer in the presence of a newly diagnosed VTE event.

Despite the variance in reported frequencies of VTE in IBD, there is an increased risk compared to the general population. Although we did not retrieve data of VTE events in patients without IBD, the VTE rates in patients with IBD in our study (1.5%) were within the range of previous well-described studies. Colonic disease in CD is considered an important risk factor. Some studies have demonstrated that an isolated colon disease is associated with increased risk [6,20], whereas others reported that colonic involvement in general was associated with higher risk [26], compatible with findings in this study (a total of 66.7% colonic and ileocolonic involvement). Complicated IBD is reported as an independent factor associated with increased risk for VTE development [6]. Although the majority of the patients who developed VTE had a non-complicated disease, 27.8% and 22.2% of patients had a stricturing and penetrating disease, respectively.

Additional figures of significance are recent hospitalizations and whether thromboprophylaxis was administered (Table S1). As stated in the results, 25% (9/36) of the newly diagnosed VTE patients were hospitalized in the prior 14 days. In accordance with a recent study that demonstrated that the risk for VTE readmission was highest in the first 10 days after hospitalization discharge, we showed a median of 8 days between hospitalizations [13]. Moreover, 77.8% of the patients with recent hospitalizations were not given prophylactic treatment at all, despite obvious risk factors (peri-operative state, IBD exacerbation, infections). These figures demonstrate the importance of a recent hospitalization as a potential risk factor, not just the current hospitalization.

As described, the consensus in hospitalized patients with IBD is to consider prophylactic anticoagulation therapy, specifically in active IBD. Of all IBD-related hospitalizations in this study, merely 12.4% (264/2137) received thromboprophylaxis. Furthermore, 38.4% (5/13) of all patients who developed VTEs during hospitalization (excluding patients who were treated with therapeutic anticoagulation) received thromboprophylaxis prior to VTE diagnosis. The decision to administer thromboprophylaxis was unrelated to their IBD status. Interestingly, they developed VTEs despite a median of 16 patient days on thromboprophylaxis, coming to emphasize the importance of additional risk factors such as immobilization, post-operative state, infections and malignancy. There was no significant difference compared to treatment rates in non IBD-related hospitalizations, as 10.9% (199/1830) received thromboprophylaxis.

Notably, there is a large variation in reported rates of VTE prophylaxis for patients with IBD in the literature [20,21]. The rates generally depend on the clinical scenario as increased prophylaxis rates can be seen in the presence of additional VTE risk factors, extensive colitis or admission to general surgery departments [22]. A recent retrospective cross-sectional study reported lower thromboprophylaxis rates (6.8%) and higher rates (1.76%) of VTE events among hospitalized IBD patients as compared to the current study [27]. This could be the result of the fact that the authors opted to exclude from their series IBD patients with known hypercoagulable risk factors.

In this study, thromboprophylaxis rates for IBD-related and non IBD-related hospitalizations were both very low, despite being conducted in a tertiary center setting. This is probably due to a number of reasons: gastroenterology consultation is not often done, especially when the hospitalization is non IBD-related; most decisions regarding the management of hospitalized patients are carried out by residents and attending physicians in the departments who lack awareness of this topic; the accepted guidelines emerged relatively late in our study period [18,19]. The latter is reflected appropriately in Figure 2, as there is a modest increment in prophylaxis rates throughout the years of this study. These unsatisfactory prophylaxis rates can have major implications, possibly life-threatening. Although there was only one death during a VTE-associated hospitalization, there was an increase when observing the following 30 and 90 days post-discharge. These incidents, as mentioned, occurred after a non IBD-related hospitalization in patients with severe medical conditions such as a metastatic malignancy and sepsis. The contribution of non-active IBD as a risk factor may possibly be less detrimental for post-hospitalization death as opposed to the combination of several devastating risk factors.

It is still debatable whether to prophylactically treat outpatients during an active IBD episode or patients discharged after an IBD-related hospitalization. As mentioned in the literature and demonstrated in our study, the majority of VTE events in patients with IBD occur as outpatients. The Canadian guidelines advocate for the treatment of IBD outpatients with moderate to severe IBD flares, assuming they have a history of a VTE provoked by an IBD flare or an unprovoked VTE [18]. Notwithstanding, in outpatients with an IBD flare without a prior VTE, there is a strong recommendation against thromboprophylaxis. In addition, the duration of treatment is inconclusive as it is based on the presence of risk factors. In terms of risk versus benefit for thromboprophylaxis, it seems the former is of greater significance. In addition, treating all outpatients may not be cost-effective, unless treatment is preserved for acute flares in the presence of high risk VTE acquired risk factors [28].

Our study has several limitations. Although we were able to analyze more than 7000 ED visits and almost 4000 hospitalizations, the low rate of VTEs diagnosed during hospitalization did not allow for multivariate analysis such as the prediction of VTE development or the efficiency of thromboprophylaxis among hospitalized patients. A much larger population-based cohort will be required for this purpose due to the fact that VTE events are still quite rare in our patient population. Second, IBD-related and non IBD-related ED visits were classified based on presenting symptoms; however, we cannot completely rule out that non IBD-related symptoms were actually due to IBD manifestations. Finally, some data were missing for patients discharged from the ED, a limitation which is often inherent to all retrospective studies.

## 5. Conclusions

In conclusion, in a large cohort of patients with IBD, we found low but non-negligible rates of VTE incidence during an IBD-related and a non IBD-related hospitalization. Furthermore, adherence and administration of thromboprophylaxis in hospitalized patients with IBD was low, despite well-known risk factors, possible implications and established guidelines, underscoring the need for better physician education and protocols implementations. Although thromboprophylaxis is recommended for hospitalized patients with IBD flares, it should be considered for non IBD-related hospitalizations as well.

## Figures and Tables

**Table 1:** Summary of ED visits and hospitalization-related factors in patients with IBD.

ED Visits, *n*	7009
Total patients, *n*	2405
Visits per patient, average (SD)	2.91 (± 5.3)
Male, *n* (% of total patients) Female, *n* (% of total patients)	1216 (50.5) 1189 (49.5)
Crohn’s disease, *n* (% of total patients)	1556 (64.7)
Ulcerative colitis, *n* (% of total patients)	849 (35.3)
Age of admission, median years (IQR)	42 (28–65)
IBD-related complaints, *n* (% of ED visits)	3305 (47.2)
Total hospitalizations, *n* (% of ED visits)	3967 (56.6)
Hospitalization unit, *n* (% of total hospitalizations)	
General surgery	1510 (38.1)
Internal medicine and geriatrics	1505 (37.9)
Short-term hospitalization unit	297 (7.5)
Pediatrics	226 (5.7)
Cardiology	76 (1.9)
Others (Orthopedics, Urology, Oncology, Gynecology)	353 (8.9)
Hospitalization length in days, median (IQR)	4 (2–7)
Return to ED in 30 days (% of ED visits)	1303 (18.6)
VTE prophylaxis, *n* (% of total hospitalizations)	463 (11.7)

ED, emergency department; IBD, inflammatory bowel disease; IQR, interquartile range; SD, standard deviation; VTE, venous; thromboembolism.

**Table 2:** Clinical characteristics of patients with IBD with newly diagnosed VTE.

Patients with Newly Diagnosed VTE	36
ED diagnosis, *n* (% of VTE)	19 (52.8)
Hospitalization diagnosis, *n* (% of VTE)	17 (47.2)
Male, *n* (%)	18 (50)
Female, *n* (%)	18 (50)
Single VTE, *n* (% of patients)	30 (83.3)
Double VTE, *n* (% of patients)	6 (16.6)
DVT, *n* (%)	34 (94.4)
PE, *n* (%)	8 (22.2)
DVT location, *n* (% of DVTs)	
Proximal	28 (82.4)
Distal	9 (26.5)
Age at onset of VTE, median (IQR)	64.5 (48.25–79.25)
Additional thrombosis-related risk factors, *n* (%)	
Malignancy	6 (16.7)
Post-operative period	6 (16.7)
Bedridden	4 (12.5)
Central venous access	2 (5.5)
No risk factors	21 (58.3)
CD, *n* (%)	18 (50)
UC, *n* (%)	18 (50)
CD extent, *n* (% of CD patients)	
L1 (ileal)	3 (16.6)
L2 (colonic)	2 (11.1)
L3 (ileo-colonic)	10 (55.6)
CD behavior, *n* (% of patients with CD)	
B1 (non-stricturing and non-penetrating)	7 (38.9)
B2 (stricturing)	5 (27.8)
B3 (penetrating)	4 (22.2)
UC extent, *n* (% of patients with UC)	
E1 (proctitis)	1 (33.3)
E2 (left sided colitis)	2 (66.7)
E3 (right sided colitis)	0 (0)
Additional extra-intestinal manifestations	8 (22.2)
Current IBD therapy, *n* (%)	
5-ASA derivatives	18 (50)
Steroids	8 (22.2)
Biologics	7 (19.4)
Immunomodulators	11 (30.6)

ASA, aminosalicylates; CD, Crohn’s disease; ED, emergency department; GI, gastrointestinal; IBD, inflammatory bowel disease; IQR, interquartile, range; UC, ulcerative colitis; VTE, venous thromboembolism.

**Table 3:** Newly diagnosed VTE during hospitalization.

IBD-Related Diagnosis, *n* (% of hospitalization VTE)	6 (35.3)
Hospitalization length, median (IQR)	21 (7–42.5)
Hospitalization unit, *n* (%)	
Internal medicine and geriatrics	11 (64.7)
General surgery	5 (29.4)
Orthopedics	1 (5.9)
Mortality, *n* (% of hospitalization VTE)	
In-hospitalization	1 (5.9)
30 days post-discharge	1 (5.9)
90 days post-discharge	1 (5.9)
Anticoagulation, *n* (% of hospitalization VTE)	
Prophylaxis	5 (29.4)
Therapeutic	4 (23.5)
None	8 (47.1)

IBD, inflammatory bowel disease; VTE, venous thromboembolism; IQR, interquartile range.

## Supplementary Materials

The following are available online at https://www.mdpi.com/2077-0383/9/9/2753/s1, Table S1: Recent hospitalizations of current VTE patients.
