# Supplementary material for: Thromboprophylaxis for Hospitalized Patients with Inflammatory Bowel Disease—Are We There Yet?
Source: J Clin Med. 2020 Aug 26;9(9):2753. doi: 10.3390/jcm9092753 (PMC7565590; doi:10.3390/jcm9092753)
Supplement: Supplementary file 1 [file jcm-09-02753-s001.pdf]

Supplementary table 1 – Recent hospitalizations of current VTE patients.

Hospitalization diagnosis		Prophylaxis	Days from last hospitalization
IBD-related, n	3		
• UC exacerbation	1	Y	6
• Small bowel obstruction	1	N	4
• Diarrhea	1	N	14
Non IBD-related, n	6		
• Aspiration pneumonia	1	Y	7
• Hypoglycemia	1	N	2
• Upper respiratory tract infection	1	N	13
• Acute renal failure	1	N	8
• Urgent colectomy (colorectal carcinoma)	2	N	11, 14

IBD – inflammatory bowel disease, UC - ulcerative colitis, VTE – venous thromboembolism
Y – yes, N- no.
